# The Neuropeptide Alpha-Melanocyte-Stimulating Hormone Is Critically Involved in the Development of Cytotoxic CD8^+^ T Cells in Mice and Humans

**DOI:** 10.1371/journal.pone.0008958

**Published:** 2010-02-01

**Authors:** Karin Loser, Thomas Brzoska, Vinzenz Oji, Matteo Auriemma, Maik Voskort, Verena Kupas, Lars Klenner, Cornelius Mensing, Axel Hauschild, Stefan Beissert, Thomas A. Luger

**Affiliations:** 1 Department of Dermatology, University of Münster, Münster, Germany; 2 Interdisciplinary Center of Clinical Research, University of Münster, Münster, Germany; 3 Department of Dermatology, University of Kiel, Kiel, Germany; New York University, United States of America

## Abstract

**Background:**

The neuropeptide alpha-melanocyte-stimulating hormone is well known as a mediator of skin pigmentation. More recently, it has been shown that alpha-melanocyte-stimulating hormone also plays pivotal roles in energy homeostasis, sexual function, and inflammation or immunomodulation. Alpha-melanocyte-stimulating hormone exerts its antiinflammatory and immunomodulatory effects by binding to the melanocortin-1 receptor, and since T cells are important effectors during immune responses, we investigated the effects of alpha-melanocyte-stimulating hormone on T cell function.

**Methodology/Principal Findings:**

T cells were treated with alpha-melanocyte-stimulating hormone, and subsequently, their phenotype and function was analyzed in a contact allergy as well as a melanoma model. Furthermore, the relevance of alpha-melanocyte-stimulating hormone–mediated signaling for the induction of cytotoxicity was assessed in CD8^+^ T cells from melanoma patients with functional and nonfunctional melanocortin-1 receptors. Here we demonstrate that the melanocortin-1 receptor is expressed by murine as well as human CD8^+^ T cells, and we furthermore show that alpha-melanocyte-stimulating hormone/melanocortin-1 receptor–mediated signaling is critical for the induction of cytotoxicity in human and murine CD8^+^ T cells. Upon adoptive transfer, alpha-melanocyte-stimulating hormone–treated murine CD8^+^ T cells significantly reduced contact allergy responses in recipient mice. Additionally, the presented data indicate that alpha-melanocyte-stimulating hormone via signaling through a functional melanocortin-1 receptor augmented antitumoral immunity by up-regulating the expression of cytotoxic genes and enhancing the cytolytic activity in tumor-specific CD8^+^ T cells.

**Conclusions/Significance:**

Together, these results point to an important role of alpha-melanocyte-stimulating hormone in MHC class I-restricted cytotoxicity. Therefore, treatment of contact allergies or skin cancer with alpha-melanocyte-stimulating hormone or other more stable agonists of melanocortin-1 receptor might ameliorate disease or improve antitumoral immune responses.

## Introduction

The neuropeptide alpha-melanocyte-stimulating hormone (α-MSH) is derived from the precursor protein pro-opiomelanocortin (POMC) by proteolytic cleavage in several cell types like melanocytes, keratinocytes, epithelial cells, B cells, natural killer cells and subsets of T cells [1]–[3]. Although originally discovered in the pituitary gland and named for its effects on pigmentation more recent studies revealed that α-MSH interacts with different types of cells such as macrophages, neutrophils or epidermal cells resulting in a broad spectrum of functions including anti-microbial, anti-inflammatory, as well as immunomodulatory activities [4]–[9].

Alpha-MSH exerts its biological function through binding to one of five different melanocortin receptors (MC-R) termed MC-1R to MC-5R. MC-R are G-protein coupled receptors with seven-transmembrane domains transmitting their effects into the cell by activating adenylate cyclase resulting in an increase of intracellular cAMP [10]–[12]. MC-1R was the first melanocortin receptor that has been cloned from melanocytes it has been demonstrated that MC-1R plays an important role in the regulation of melanogenesis and pigmentation since functional mutations of this receptor greatly impact fur color in mammals as well as hair and skin color in humans [10], [13], [14]. Moreover, MC-1R has been suggested to be critically involved in melanoma susceptibility since certain mutations in the *MC-1R* gene are strongly associated with increased melanoma incidence by sensitizing melanocytes to the cytotoxic effects of UV irradiation [15].

However, the observation that MC-1R is also present on non-melanocytic cutaneous cells, mucosal cells of the gastrointestinal tract as well as various cells of the immune system including macrophages, dendritic cells (DC), mast cells, and neutrophils rather pointed to a broader role of MC-1R in inflammation and immunity [16], [17]. Among all melanocortin receptors MC-1R has the highest affinity to α-MSH and it has therefore been suggested that many of the anti-inflammatory and immunomodulatory activities of α-MSH are mediated through binding to and activation of MC-1R [18], [19].

The molecular mechanisms underlying the immunomodulatory activities of α-MSH are not yet completely understood. However, inhibition of nuclear transcription factor-κB (NFκB) activation and protection against IκBα degradation finally leading to reduced production of pro-inflammatory cytokines seem to be crucial for the α-MSH mediated effects on immune responses as evidenced by *in vitro* studies in different cell types [4], [19]–[21]. Furthermore, it has been shown that α-MSH also exhibits immunomodulatory effects *in vivo.* In a mouse model, Grabbe et al. demonstrated that systemic as well as epicutaneous application of α-MSH suppressed the sensitization and elicitation phase of contact allergy and induced a hapten-specific tolerance [22]. In addition, systemic administration of α-MSH efficiently inhibited the development of dextran sodium sulphate induced colitis, experimental autoimmune encephalomyelitis or allergic airway inflammation in mice as well as adjuvant-induced arthritis or experimental uveitis in rats [23]–[27]. Based on these observations a potential role for α-MSH in the regulation of the immune responses seems to be very likely. This assumption is further strengthened by the fact that various cells of the immune system express POMC and have been shown to release increased levels of α-MSH during inflammation [28].

Since T cells are important effectors during immune responses we have investigated the effects of α-MSH on T cell function. Here we demonstrate that MC-1R is expressed by murine as well as human CD8^+^ T cells and furthermore, we show that α-MSH/MC-1R mediated signaling is important for the induction of cytotoxicity in human and murine CD8^+^ T cells. Upon adoptive transfer α-MSH treated murine CD8^+^ T cells significantly reduced contact allergy responses in recipient mice. Additionally, the presented data indicate that α-MSH augmented anti-tumoral immunity by up-regulating the expression of cytotoxic genes and enhancing the cytolytic activity in tumor-specific CD8^+^ T cells.

## Results

### MC-1R Is Expressed in Murine CD8+ T Cells

**Figure 1:**
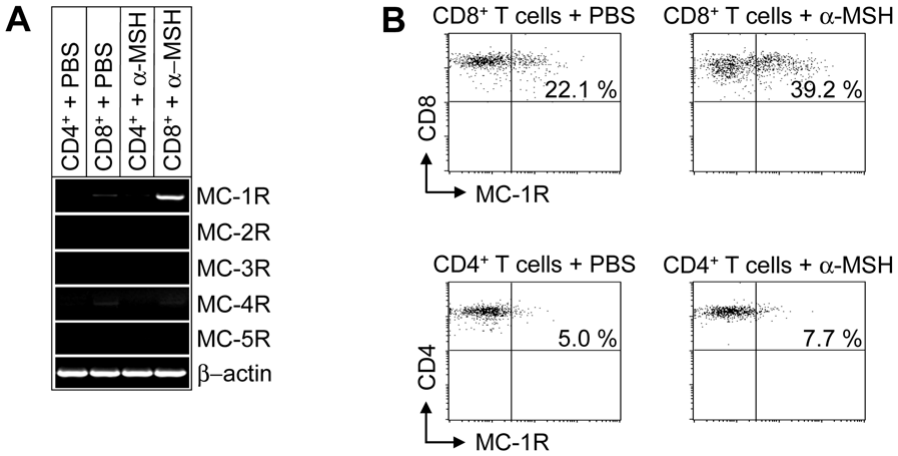
MC-1R is expressed on murine CD8^+^ T cells and up-regulated after stimulation with α-MSH. (**A**) RT- PCR analysis of CD4^+^ and CD8^+^ T cells after stimulation with α-MSH or PBS. (**B**) FACS analyses of CD8^+^ and CD4^+^ T cells from wt mice (total number of animals analyzed n = 15) after stimulation of cells with PBS or α-MSH. Representative dot blots are shown for each experimental group and cells are gated for CD8 and CD4, respectively.

To study the effects of α-MSH on T cells, we analyzed the expression of MC-Rs in CD4^+^ and CD8^+^ T cells. Whereas MC-2R, MC-3R, MC-4R and MC-5R are not expressed by T cells, MC-1R was found in naive murine CD8^+^ T cells and was up-regulated upon stimulation with α-MSH (Figure 1A). In contrast, MC-1R mRNA was not detectable in CD4^+^ T cells (Figure 1A). These results were further confirmed by flow cytometry. As shown in Figure 1B, MC-1R was detectable in a subpopulation of naive CD8^+^ T cells and stimulation with α-MSH significantly increased the number of MC-1R expressing CD8^+^ T cells. However, only a feeble expression of MC-1R was detectable in naive CD4^+^ T cells and moreover, no up-regulation was found after stimulation with α-MSH (Figure 1B).

### CD8+ T Cells Stimulated with α-MSH Reduce Contact Allergy

**Figure 2:**
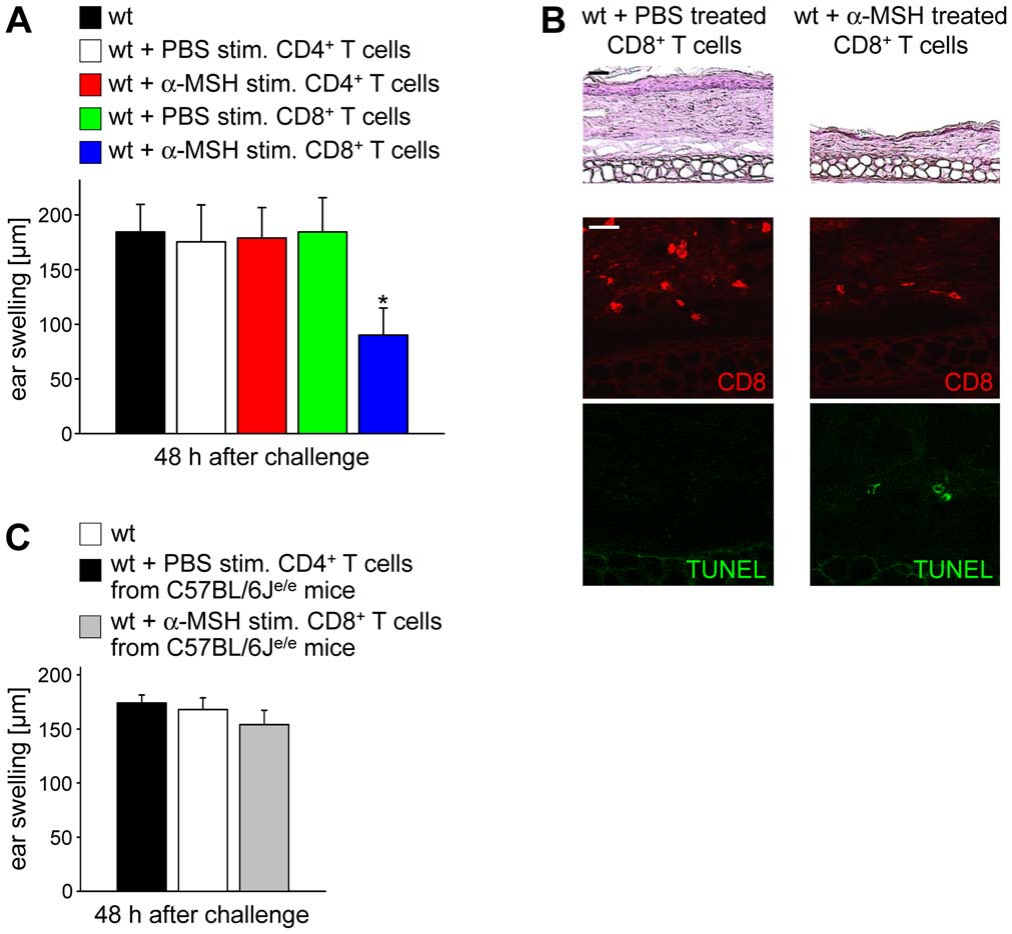
Alpha-MSH stimulated CD8^+^ T cells from wt mice suppress contact allergy responses. (**A**) Twenty-four h before challenge, DNFB-sensitized recipients were injected intravenously with 5×10^6^ CD8^+^ T cells from wt donors that had been stimulated with PBS or α-MSH and co-cultured with DNBS-pulsed DC prior to adoptive cell transfer. Data are shown as mean ear swelling ± SD and are representative of 15 mice in three independent experiments. * *P*<0.05 vs. transfer of PBS stimulated CD8^+^ T cells. (**B**) Lymphocyte infiltrations and apoptotic cell death were determined by H&E (original magnification: 200×, scale bar  = 25 µm) or immunofluorescence staining of ear skin (original magnification: 300×, scale bar  = 25 µm). (**C**) MC-1R mediated signaling in CD8^+^ T cells is required for the suppression of contact allergy. Twenty-four h before challenge, recipients were injected intravenously with 5×10^6^ PBS or α-MSH stimulated CD8^+^ T cells from C57BL/6J^e/e^ mice. Data are shown as mean ear swelling ± SD and are representative of 10 mice.

Since MC-1R is expressed on murine CD8^+^ T cells we speculated that α-MSH might directly affect the phenotype or function of CD8^+^ T cells by binding to MC-1R. To investigate the effects of α-MSH on CD8^+^ T cells *in vivo* we performed contact allergy experiments since contact allergy responses are predominantly mediated by CD8^+^ T cells [29]. Therefore, CD8^+^ T cells were stimulated with α-MSH or PBS, co-cultured with 2,4-dinitrobenzene sulfonic acid (DNBS)-pulsed bone marrow derived DC (bmDC), and intravenously injected into 2,4-dinitro-1-fluorobenzene (DNFB)-sensitized recipient mice. Subsequently, recipients were ear challenged and the ear swelling was assessed. Whereas mice injected with PBS treated CD8^+^ T cells mounted normal ear swelling responses, recipients of α-MSH stimulated CD8^+^ T cells developed a significantly reduced allergic hypersensitivity (Figure 2A). In contrast, injection of α-MSH stimulated CD4^+^ T cells did not affect contact allergy responses. Moreover, reduced numbers of CD8^+^ T cells infiltrated the ears of mice injected with α-MSH stimulated CD8^+^ T cells as evidenced by immunofluorescence stainings (Figure 2B). Interestingly, we detected increased levels of apoptotic cells in the ears of mice injected with α-MSH stimulated CD8^+^ T cells whereas no TUNEL^+^ apoptotic cells were present in ear skin from recipients of PBS stimulated CD8^+^ T cells (Figure 2B). Of note, the TUNEL staining did not co-localize with CD8 (data not shown) suggesting that α-MSH might induce or up-regulate cytotoxicity in CD8^+^ T cells leading to apoptotic cell death of skin-infiltrating mononuclear cells finally resulting in decreased contact allergy responses in recipients of α-MSH stimulated CD8^+^ T cells.

To investigate whether binding of α-MSH to MC-1R was required for the reduction of contact allergy CD8^+^ T cells from C57BL/6J^e/e^ mice were stimulated with α-MSH, co-cultured with DNBS-pulsed DC, injected intravenously into DNFB-sensitized wt mice and subsequently, recipients were ear challenged to the hapten. Strikingly, adoptive transfer of α-MSH stimulated CD8^+^ T cells lacking a functional MC-1R did not reduce contact allergy responses (Figure 2C). Furthermore, we did not observe increased numbers of TUNEL^+^ apoptotic cells in recipients of α-MSH stimulated CD8^+^ T cells from C57BL/6J^e/e^ mice (data not shown) clearly indicating that binding to MC-1R is necessary for the immunomodulatory effects of α-MSH on CD8^+^ T cells.

### Increased Cytotoxicity in CD8+ T Cells Stimulated with α-MSH

**Figure 3:**
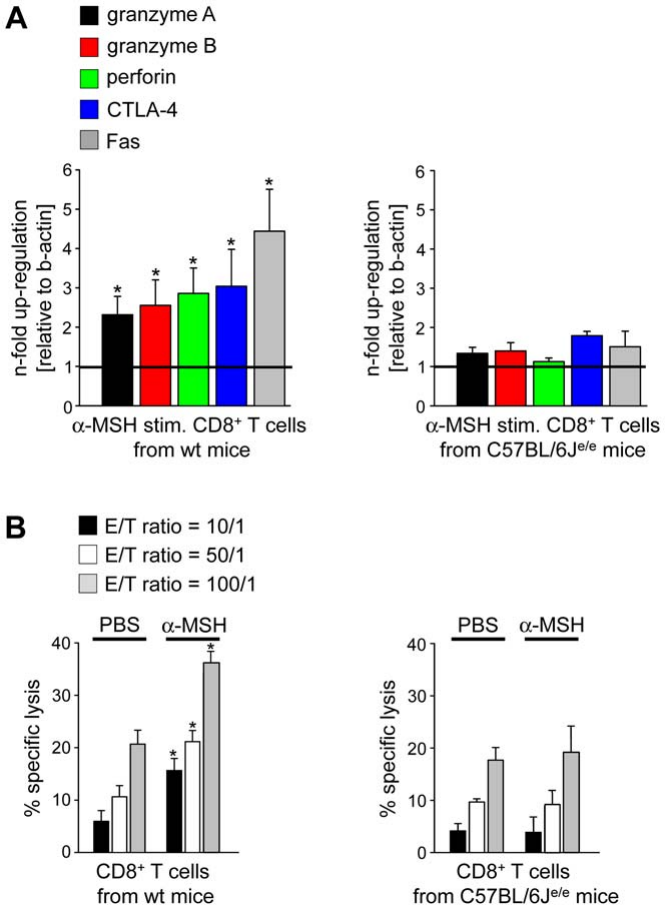
Alpha-MSH/MC-1R signaling induces cytotoxicity in CD8^+^ T cells. (**A**) CD8^+^ T cells were isolated from wt or C57BL/6J^e/e^ mice and stimulated with PBS or α-MSH. Subsequently, real-time PCR analyses were performed and the relative mRNA expression levels of granzyme A, granzyme B, perforin, CTLA-4, and Fas in α-MSH stimulated CD8^+^ T cells vs. PBS treated controls are shown (gene expression in PBS treated CD8^+^ T cells  = 1). * *P*<0.05 vs. PBS stimulated CD8^+^ T cells. (**B**) Increased cytolytic activity in α-MSH stimulated CD8^+^ T cells. CD8^+^ T cells were purified from wt or C57BL/6J^e/e^ mice and stimulated with PBS or α-MSH. Subsequently, the cytotoxic activity of CD8^+^ T cells was analyzed 6 h after co-culture with chromium-loaded L1210/3 target cells. One representative out of three independent experiments is shown. * *P*<0.05 vs. PBS stimulated CD8^+^ T cells.

To analyze whether the reduction of contact allergy as well as the increased numbers of apoptotic cells in skin biopsies from recipients of α-MSH stimulated CD8^+^ T cells (Figures 2A, 2B) could indeed be attributed to increased cytotoxicity we performed gene expression studies. Therefore, CD8^+^ T cells from wt and C57BL/6J^e/e^ mice were stimulated with PBS or α-MSH. Interestingly, the relative mRNA levels of genes directly associated with cytotoxicity of CD8^+^ T cells such as granzyme A, granzyme B, perforin, Fas (CD95) or CTLA-4 (CD152) were significantly enhanced in α-MSH stimulated CD8^+^ T cells from wt mice compared to PBS treated controls (Figure 3A) suggesting that α-MSH up-regulated the cytotoxic potential of CD8^+^ T cells. Of note, the α-MSH mediated induction of cytotoxicity in CD8^+^ T cells was dependent on melanocortin signaling via a functional MC-1R since α-MSH treatment of CD8^+^ T cells from C57BL/6J^e/e^ mice did not induce the expression of cytotoxic genes (Figure 3A).

Next we intended to assess whether the up-regulated expression of cytotoxic genes in α-MSH stimulated CD8^+^ T cells accounted for an increased kill activity. Hence, we performed chromium release assays using allogeneic DC to activate the α-MSH stimulated CD8^+^ T cells and ^51^Cr loaded L1210/3 leukemia cells as targets. As shown in Figure 3B α-MSH stimulated CD8^+^ T cells from wt mice exhibited a significantly enhanced cytolytic activity compared to PBS treated controls. However, binding of α-MSH to a functional MC-1R was an important prerequisite for the induction of kill activity since the cytotoxic potential of α-MSH stimulated CD8^+^ T cells from C57BL/6J^e/e^ mice was not altered compared to negative controls (Figure 3B).

### Reduced Tumor Growth in Mice Injected with α-MSH Treated CD8+ T Cells

**Figure 4:**
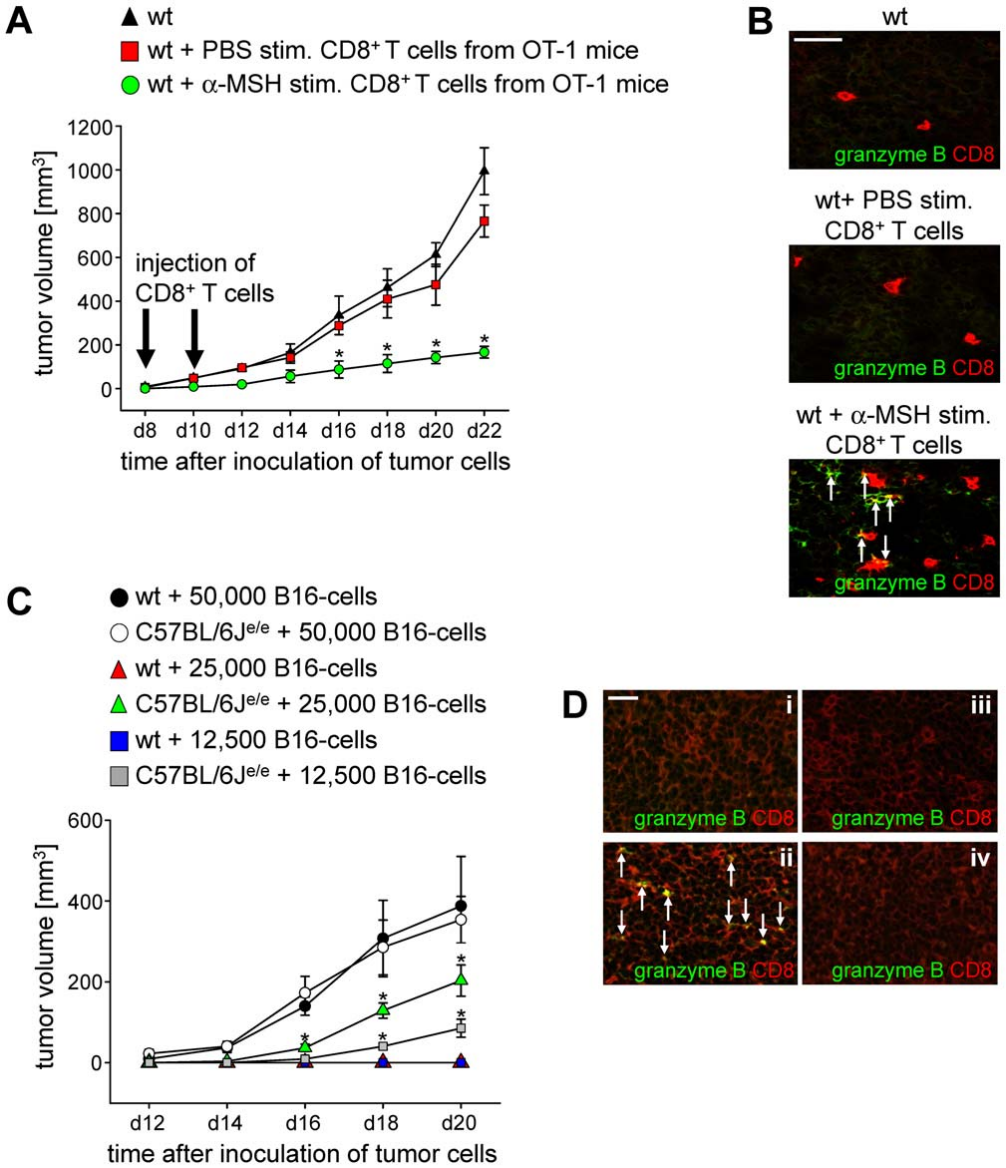
Reduced tumor growth in mice injected with α-MSH treated CD8^+^ T cells. (**A**) Wt mice were inoculated with 1×10^5^ B16-OVA cells and injected with 5×10^6^ PBS or α-MSH stimulated CD8^+^ T cells from OT-1 mice at d8 and d10 after inoculation of tumor cells. Tumor growth was monitored over time in n = 18 mice in three independent experiments. * *P*<0.05 vs. transfer of PBS stimulated CD8^+^ T cells. (**B**) Immunofluorescence staining of tumor tissue at d22 after inoculation of tumor cells using antibodies directed against CD8 and granzyme B. Original magnification: 400×, scale bar  = 25 µm. Cells expressing CD8 and granzyme B are marked by arrows. (**C**) MC-1R signaling is important for MHC class I-restricted anti-tumoral immunity. Wt and C57BL/6J^e/e^ mice were inoculated with indicated concentrations of B16 cells and tumor growth was monitored over time. One representative out of three independent experiments is shown. * *P*<0.05 vs. wt. (**D**) Immunofluorescence staining of contralateral and tumor-draining lymph nodes from wt mice (i and ii) as well as C57BL/6J^e/e^ mice (iii and iv) at d20 after inoculation of 25,000 B16 cells using antibodies directed against CD8 and granzyme B. Original magnification: 300×, scale bar  = 25 µm. Cells expressing CD8 and granzyme B are marked by arrows.

Since cytotoxic CD8^+^ T cells play an important role in anti-tumoral immunity [30] we investigated whether the cytolytic activity of tumor-antigen specific cytotoxic CD8^+^ T cells could be increased by stimulation with α-MSH. To address this, groups of wt mice were inoculated with 1×10^5^ B16-OVA melanoma cells. At day 8 and day 10 after inoculation of tumor cells mice were injected intravenously with either 5×10^6^ PBS- or α-MSH stimulated CD8^+^ T cells from OT-1 mice. Strikingly, α-MSH stimulated CD8^+^ T cells significantly reduced progressive tumor growth in the recipient mice in contrast to PBS treated controls (Figure 4A) strongly indicating that α-MSH indeed induced cytotoxic CD8^+^ T cells that were functional *in vitro* as well as *in vivo*. These results were further confirmed by immunofluorescence stainings of tumor tissue at d22 after inoculation of B16-OVA cells. Whereas we detected only low frequencies of CD8^+^ T cells that did not express cytotoxic markers in the tumors of recipients treated with PBS stimulated CD8^+^ cells, a substantial amount of CD8^+^ T cells expressing granzyme B infiltrated the tumors of recipient mice that were injected with α-MSH stimulated CD8+ T cells (Figure 4B). Of note, in mice injected with α-MSH stimulated CD8^+^ T cells from C57BL/6J^e/e^ mice we did neither observe a reduction in tumor growth nor an increased infiltration of granzyme B expressing CD8^+^ T cells into the tumors (data not shown) again demonstrating that α-MSH/MC-1R signaling is required for the induction of cytotoxic gene expression and cytolytic activity in CD8^+^ T cells.

Since α-MSH via binding to MC-1R up-regulated cytotoxicity in tumor-specific CD8^+^ T cells we speculated that C57BL/6J^e/e^ mice might exhibit impaired anti-tumoral immunity. To analyze this hypothesis wt and C57BL/6J^e/e^ mice were inoculated with different concentrations of B16-melanoma cells. Interestingly, inoculation of C57BL/6J^e/e^ mice with 25,000 or 12,500 tumor cells still resulted in progressive tumor growth whereas we did not observe solid tumors in wt mice after injection of these cell numbers (Figure 4C) indicating that α-MSH/MC-1R signaling plays an important role for anti-tumoral immune responses *in vivo* by inducing/up-regulating cytotoxic activity in CD8^+^ T cells. This induction of cytotoxicity was associated with the increased expression of genes characteristic for the function of cytotoxic T lymphocytes since we detected granzyme B^+^CD8^+^ T cells in tumor-draining lymph nodes of wt mice injected with 25,000 tumor cells whereas CD8^+^ T cells expressing cytotoxic markers were neither detectable in tumor-draining nor contra-lateral lymph nodes of C57BL/6J^e/e^ mice (Figure 4D).

**Figure 5:**
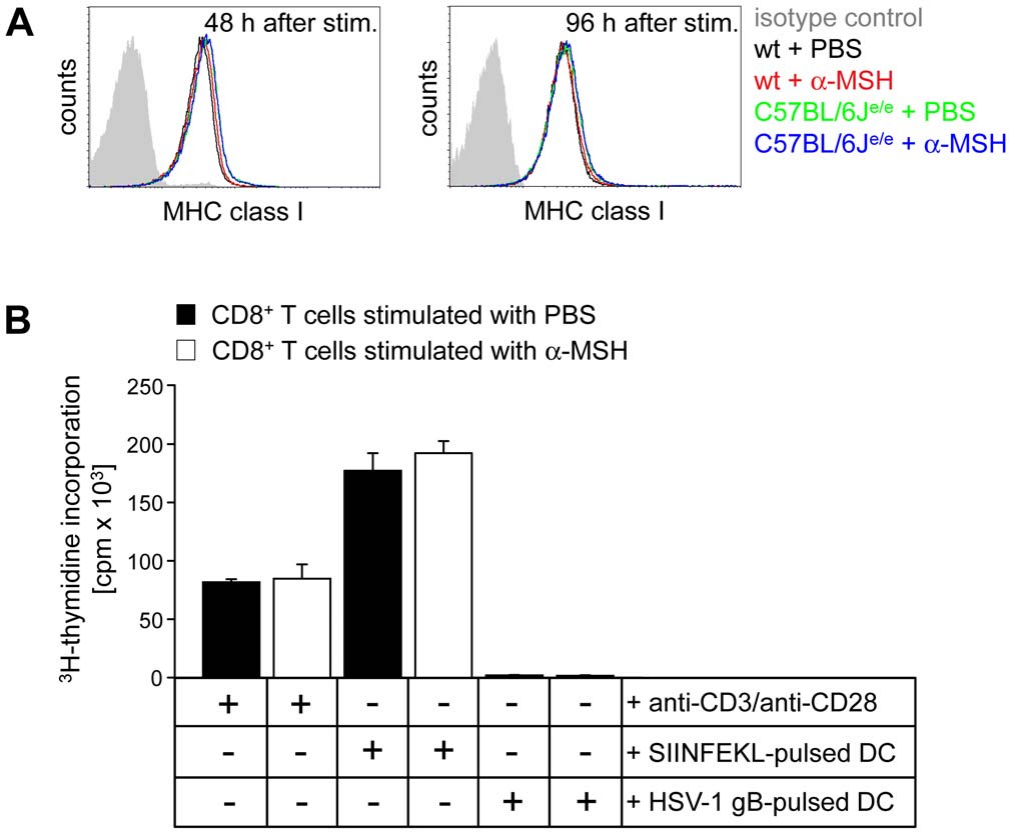
Alpha-MSH does not influence MHC I expression in CD8^+^ T cells or the capacity to recognize antigens. (**A**) Flow cytometric analysis of CD8^+^ T cells from wt or C57BL/6J^e/e^ mice (n = 5 mice each group) after stimulation with α-MSH (10^−9^ molar) for 48 h or 96 h. Cells were gated for CD8 and MHC class I expression is shown in a representative histogram overlay. (**B**) PBS and α-MSH stimulated CD8^+^ T cells from OT-1 mice equally recognize the relevant antigen presented by DC. BmDC were pulsed with SIINFEKL or HSV-1 gB peptides and co-cultured with PBS or α-MSH stimulated CD8^+^ T cells from OT-1 mice for 3 days (DC/T cell ratio  = 1/40). Subsequently, T cell proliferation was assessed by ^3^H-thymidine incorporation. One representative experiment out of three is shown.

It has been suggested that α-MSH might be able to down-regulate MHC class I expression in melanoma cell lines [31]. Hence, we analyzed whether α-MSH also affects the MHC class I expression in purified CD8^+^ T cells, a molecule needed to recognize and respond to relevant tumor-antigens. Therefore, CD8^+^ T cells were stimulated with α-MSH in a concentration of 10^−9^ molar for 48 h and 96 h and subsequently, the expression of MHC class I was quantified by flow cytometry. As shown in Figure 5A MHC class I expression was identical in α-MSH stimulated CD8^+^ T cells compared to non-treated controls. Moreover, MHC class I expression was not altered in α-MSH stimulated CD8^+^ T cells from C57BL/6J^e/e^ mice compared to CD8^+^ T cells from wt controls indicating that α-MSH/MC-1R mediated signaling does not affect the MHC class I expression in CD8^+^ T cells (Figure 5A). Furthermore, α-MSH did not affect the capacity of CD8^+^ T cells to recognize and respond to antigens since CD8^+^ T cells from OT-1 mice that have been stimulated with α-MSH showed a similar proliferation upon co-culture with SIINFEKL-pulsed DC compared to PBS-treated controls (Figure 5B).

**Figure 6:**
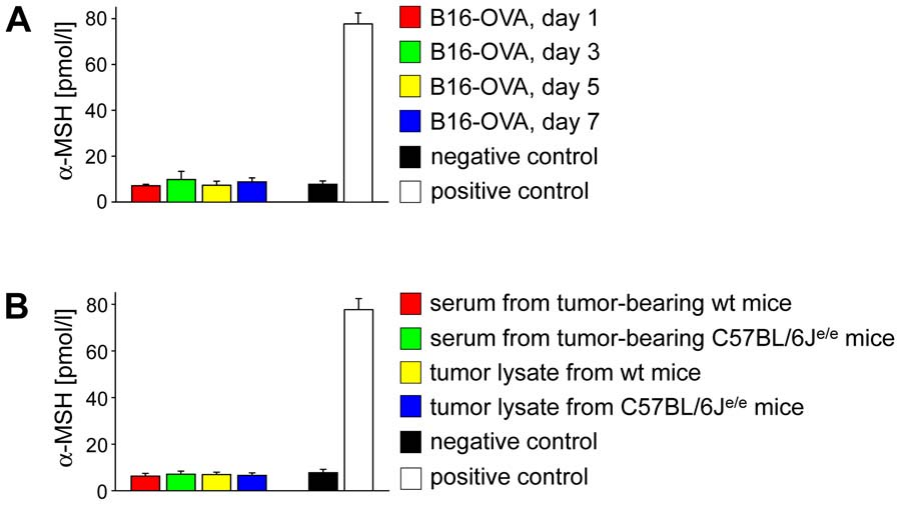
Alpha-MSH is not secreted by B16-OVA melanoma cells or up-regulated in mice bearing B16-OVA tumors. (**A**) Alpha-MSH was quantified in culture supernatants from B16-OVA melanoma cells at indicated time points by radioimmunoassays. (**B**) Alpha-MSH was quantified in the serum as well as in tumor lysates from tumor-bearing wt and C57BL/6J^e/e^ mice 21 days after subcutaneous injection of 100,000 B16-OVA melanoma cells (n = 5 mice each group).

As α-MSH was reported to induce immunosuppression [22] we intended to analyze whether the ovalbumin transfected melanoma cell line B16-OVA secreted detectable levels of α-MSH. Thus, we quantified α-MSH in culture supernatants from B16-OVA cells at d1, d3, d5 and d7 of cell culture by radioimmunoassay. As shown in Figure 6A B16-OVA cells did not secrete α-MSH *in vitro*. To exclude that the α-MSH secretion was exclusively induced *in vivo* we injected B16-OVA cells into the flanks of wt and C57BL/6J^e/e^ mice and quantified the α-MSH concentration in the sera of the mice as well as in tumor lysates 3 weeks after inoculation of tumor cells. The data presented in Figure 6B clearly demonstrate that α-MSH secretion in B16-OVA cells was not induced *in vivo* since we did neither detect systemically increased α-MSH levels in tumor-bearing mice nor locally elevated α-MSH concentrations in the tumor tissue. Together, these data indicate that α-MSH release might play a minor role in B16-OVA melanoma cells.

### Alpha-MSH/MC-1R Signaling Plays an Important Role for the Function of Human CD8+ Cytotoxic T Cells

**Figure 7:**
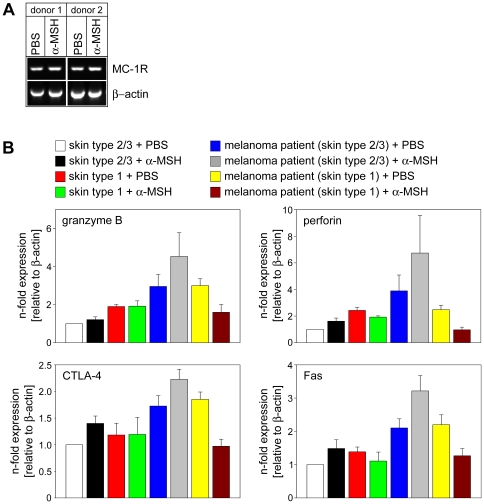
Alpha-MSH/MC-1R signaling is involved in the induction of cytotoxic activity in human CD8^+^ T cells. (**A**) MC-1R is expressed in human CD8^+^ T cells. RT- PCR of purified CD8^+^ T cells from two healthy donors after stimulation with α-MSH or PBS. (**B**) CD8^+^ T cells from healthy donors with skin type 1 or skin type 2/3 as well as melanoma patients with skin type 1 or skin type 2/3 were stimulated with PBS or α-MSH and subjected to real-time PCR analyses. Relative mRNA expression levels of granzyme B, perforin, CTLA-4, and Fas in α-MSH stimulated CD8^+^ T cells and PBS treated controls from 6–12 patients in each group are shown.

Next we were interested to investigate whether α-MSH/MC-1R signaling is involved in the regulation of cytotoxicity in human CD8^+^ T cells. First, we analyzed the expression of MC-1R in human CD8^+^ T cells. As shown in Figure 7A, MC-1R was found in naive human CD8^+^ T cells and its mRNA expression was not altered upon stimulation with α-MSH (Figure 1A). Thus, the identification of MC-1R in human CD8^+^ T cells permitted to scrutinize a role of α-MSH/MC-1R signaling for the induction of cytotoxic activity.

Since also in humans cytotoxic CD8^+^ T cells are critically involved in anti-tumoral immunity, e.g. against melanoma [32] we intended to investigate whether the cytolytic activity of tumor-specific cytotoxic CD8^+^ T cells from melanoma patients could be increased by stimulation with α-MSH. Furthermore, to assess the relevance of α-MSH/MC-1R signaling for the regulation of cytotoxicity in human CD8^+^ T cells melanoma patients with red hair and fair skin (skin type 1) were included. In humans variation of hair and skin color is regulated by MC-1R and it has been shown that loss-of-function mutations in MC-1R result in the red hair and fair skin phenotype [33], [34]. To confirm the presence of loss-of-function mutations in MC-1R we sequenced the MC-1R gene. As expected, melanoma patients and healthy donors with skin type 2 or 3 did not show any mutation (Table 1). By contrast, in the DNA from all healthy donors and melanoma patients with skin type 1 we found several mutations previously associated with the red hair and fair skin phenotype as well as the loss-of-function of MC-1R (Table 1) [35]. Interestingly, in the MC-1R genes of all melanoma patients with skin type 1 mutations were detected that have been described to correlate with skin cancer or to be directly linked to melanoma [35]–[39] whereas healthy donors with skin type 1 predominantly exhibited mutations that have only been associated with the red hair/fair skin phenotype or the loss-of-function of MC-1R (Table 1) [35], [40], [41].

**Table 1:** *MC-1R* sequencing analysis.

Sample ID	Change(s) of DNA sequence	*MC1R* genotype	Reference
i	-	*wt*/*wt*	-
ii	-	*wt*/*wt*	-
1	1868 G>A het	*R163Q*/*wt*	[35], [40]
2	1558 G>T/1654G>A	*V60L*/*V92M*	[35], [40], [41]
3	1868 G>A het	*R163Q*/*wt*	[35], [40]
A	**1467insA**/1831 C>T	*30fsX13*/***R151C***	[35]–[39]
B	1831 C>T/**2260 G>C**	***R151C***/***D294H***	[35]–[39]
C	1831 C>T hom	***R151C***/***R151C***	[35]–[39]
D	1831 C>T/**1858 C>T**	***R151C***/***R160W***	[35]–[39]
E	1831 C>T hom	***R151C***/***R151C***	[35]–[39]
F	**1805 G>A**/**1858 C>T**	*R142H*/***R160W***	[35]–[39]
G	**1805 G>A**/1831 C>T	*R142H*/***R151C***	[35]–[39]
H	1831 C>T/**1858 C>T**	***R151C***/***R160W***	[35]–[39]
I	**1805 G>A**/1831 C>T	*R142H*/***R151C***	[35]–[39]
K	**1632 C>A**/**1858 C>T**	***D84E***/***R160W***	[35]–[39]
L	1831 C>T hom	***R151C***/***R151C***	[35]–[39]
M	**1558 G>T**/**1858 C>T**	*V60L*/***R160W***	[35]–[41]

MC-1R sequence analysis of an exemplary healthy donor (skin type 2/3, i), an exemplary melanoma patient (skin type 2/3, ii), three healthy donors with red hair and fair skin (skin type 1, 1–3) and 12 melanoma patients with skin type 1 (A–M). Alleles that have been shown to be associated with the red hair/fair skin phenotype are underlined; mutations which alter the ability to bind α-MSH and/or activate adenylyl cyclase are printed in bold and are underlined. Variations of the MC-1R gene that have been associated with an increased risk for melanoma development are shown in bold.

het  =  heterozygous; hom  =  homozygous; wt  =  wildtype.

After having identified a collective of patients/donors with functional MC-1R and an equal collective with impaired MC-1R function we stimulated purified CD8^+^ T cells with α-MSH and subjected them to quantitative realtime PCR analyses. As shown in Figure 7B, the expression of genes tightly linked to the cytolytic activity of CD8^+^ T cells was significantly enhanced in PBS treated CD8^+^ T cells from metastatic melanoma patients with skin type 2/3 compared to healthy controls possibly pointing to ongoing anti-tumoral immune responses. Moreover, we detected increased granzyme B-, perforin-, Fas-, and CTLA-4 mRNA levels in α-MSH stimulated CD8^+^ T cells form melanoma patients with skin type 2/3 compared to PBS treated cells from the same patient (Figure 7B) indicating that also in human CD8^+^ T cells α-MSH up-regulated the expression of cytotoxic genes. Strikingly, α-MSH did not up-regulate the expression of cytotoxic genes in CD8^+^ T cells from melanoma patients with skin type 1 compared to PBS treated cells from the same patient suggesting that the induction of cytotoxicity in human CD8^+^ T cells requires functional MC-1R signaling. Together, these data demonstrate that α-MSH plays an important role for the induction or up-regulation of cytotoxic activity in murine as well as human CD8^+^ T cells via signaling through a functional MC-1R.

## Discussion

The neuropeptide α-MSH has been shown to exert multiple anti-inflammatory and immunomodulatory effects whereas in particular the immunomodulatory effects are mediated via binding to MC-1R [42]. Here we demonstrated that MC-1R is expressed by a subset of murine and human CD8^+^ T cells and furthermore, we show that α-MSH/MC-1R mediated signaling is crucially involved in the induction of cytotoxicity in human as well as murine CD8^+^ T cells. Additionally, we provide evidence that α-MSH augments anti-tumoral immunity by up-regulating the expression of cytotoxic genes and enhancing the cytolytic activity in tumor-specific CD8^+^ T cells.

These observations as well as its inhibitory effects on melanoma proliferation and metastasis formation [43]–[45] indicate a protective role for α-MSH in melanoma. Moreover, it has been speculated that the anti-inflammatory properties of α-MSH may also affect melanoma progression. Indeed, in the tumor microenvironment α-MSH via down-regulation of cell adhesion molecules or MHC class I expression might reduce the migratory capacity of tumor-specific T cells and the interaction between melanoma cells and cytotoxic T cells [46]–[50]. Since melanoma is one of the tumors known to frequently show spontaneous remission usually attributed to anti-tumoral immune responses as evidenced by the high number of case reports [51]–[53], an effect of α-MSH potentially facilitating the escape of melanoma from immunosurveillance might be of minor relevance. In our model α-MSH significantly increased the cytotoxic activity of CD8^+^ T cells, resulting in an effective anti-tumoral immune response. It should, however, be kept in mind that our system is an experimental model which cannot and is not intending to fully mimic the complex *in vivo* situation. By contrast, our experimental setup allows dissecting the direct effects of α-MSH on the cytolytic activity of tumor-specific CD8^+^ T cells, an important component of anti-tumoral immunity.

Another important difference between the *in vivo* situation and our melanoma model lies in the fact that we used ovalbumin as model-antigen and transgenic T cells expressing an ovalbumin-specific T cell receptor. *In vivo* cytotoxic T cells would be instructed by antigen-presenting cells and it is well accepted that α-MSH via binding to MC-R is able to modulate the function of antigen-presenting cells [3], [4], [6]. Therefore, it would be important to analyze the effects of *ex vivo* α-MSH stimulated DC on tumor growth as well as on the activation status of tumor-specific cytotoxic CD8^+^ T cells in the B16-OVA melanoma model. Moreover, the continuous depletion of α-MSH from tumor-bearing mice using neutralising antibodies could be able to provide additional data clarifying the effects of α-MSH/MC-R mediated signaling in melanoma *in vivo*.

In recent studies it has been shown that at least some types of MC-R are expressed not only in melanocytes but also in the majority of nonmelanocytic cell types including various cells of the immune system such as monocytes, neutrophils, dendritic cells, and lymphocytes [9], [16], [54]–[56]. However, the expression of MC-R in T cells has been discussed controversially. Whereas Cooper et al. found no evidence of any MC-R expression in human T cells, using multicolor flow cytometry Neumann-Andersen et al. showed MC-1R to be constitutively present on a subset of CD8^+^ T cells [55], [57]. These divergent results might be attributed to different cell culture conditions. Whereas Neumann-Andersen et al. used freshly isolated peripheral blood mononuclear cells (PBMC), the cells analyzed by Cooper et al. were cultured in the presence of streptokinase and streptodornase for 48 h prior to RNA extraction. Furthermore, both groups used different primer sequences and therefore, did not amplify identical regions of the MC-R genes [55]–[57]. The results presented here demonstrate MC-1R expression in human CD8^+^ T cells as well as in a subset of murine CD8^+^ T cells, whereas the mRNA or protein concentration was below detectable levels in CD4^+^ T cells. Hence, our results are in accordance with the earlier findings by Neumann-Andersen et al. showing the expression of MC-1R on natural killer cells and cytotoxic CD8^+^ T cells whereas only 1% of CD4^+^ T cells could be stained for MC-1R [57]. This expression pattern suggested a crucial role of MC-1R signaling in the regulation of MHC class I-restricted immunity. Indeed, our data show that signaling via MC-1R induced the expression of cytotoxic genes in murine as well as human CD8^+^ T cells and significantly up-regulated the cytolytic activity as shown in chromium release assays.

Several studies demonstrated that the immunomodulatory activities of α-MSH are predominantly mediated through binding to MC-1R [9], [16], [23], [54]. The vast majority of data supporting the relevance of α-MSH/MC-1R signaling for the immunomodulatory effects of α-MSH have been carried out *in vitro*. However, in a murine model of DSS-induced experimental colitis MC-1R has been shown to play a role in intestinal inflammation since disease progression was aggravated in C57BL/6J^e/e^ mice indicating that α-MSH/MC-1R mediated signaling is crucial for intestinal inflammation [58]. In the present study we demonstrate that α-MSH/MC-1R mediated signaling plays an important role for the regulation of cytotoxicity and anti-tumoral immunity. Alpha-MSH significantly increased the expression of genes associated with cytotoxic activity and moreover, up-regulated the cytolytic potential of CD8^+^ T cells with functional MC-1R (Figures 3A, 3B). Importantly, injection of tumor-antigen specific CD8^+^ T cells stimulated via α-MSH/MC-1R signaling into tumor-bearing recipient mice significantly reduced progressive tumor growth indicating that α-MSH/MC-1R interactions were crucial for the induction of tumor-specific cytotoxic T lymphocytes. Of note, this effect was strongly dependent on binding of α-MSH to a functional MC-1R since α-MSH stimulated CD8^+^ T cells from C57BL/6J^e/e^ mice did not exhibit cytotoxic activity. Furthermore, α-MSH/MC-1R signaling seems to be important for the induction of tumor-specific cytotoxic CD8^+^ T cells *in vivo* since in comparison to wt controls C57BL/6J^e/e^ mice inoculated with similar numbers of B16 melanoma cells showed an increased tumor growth and reduced infiltration of CD8^+^ cytotoxic T cells in tumor-draining lymph nodes (Figure 4C).

In humans, MC-1R is known to account for substantial variation in skin cancer incidence. Even when adjustments for skin phenotype are made, an influence of MC-1R on skin cancer development is still evident suggesting that the MC-1R signaling pathway might play a yet undescribed role in cutaneous biology accounting for the association of MC-1R and skin cancer [59]. Our novel findings point to the relevance of α-MSH/MC-1R signaling for the up-regulation of cytotoxic gene expression in melanoma patients which is impaired in patients with a non-functional MC-1R (Figure 7B). Furthermore, our results might represent a missing link between the yet unknown role of MC-1R in cutaneous biology and the influence of MC-1R in skin cancer development.

The *MC-1R* coding sequence is known to be highly polymorphic and associations of certain allelic variants with pigmentation phenotypes and risk factors for melanoma and non-melanoma skin cancer development have been reported frequently [35], [37], [39], [60], [61]. Currently, more than 30 variant alleles have been described for European populations whereas four alleles could be classified as having a strong association with the red hair/fair skin phenotype: D84E, R151C, R160W, and D294H. Others, such as V60L, V92M, and R163Q, only showed a weak association. Moreover, loss of function alleles, such as R151C, R160W, R142H, and D294H, which alter the ability to bind α-MSH and/or activate adenylyl cyclase, lead to increased sensitivity towards the cytotoxic effects of UV radiation and could be strongly associated with an increased risk to develop all forms of skin cancer including melanoma [62]–[68]. Interestingly, we detected loss of function mutations associated with a high incidence for skin cancer in all melanoma patients with red hair/fair skin whereas all analyzed healthy donors with skin type 1 only had allelic variations associated with the red hair/fair skin phenotype but without significant association to skin cancer (Table 1).

Based on these observations and based on our findings that functional α-MSH/MC-1R signaling up-regulates the expression of cytotoxic genes in CD8^+^ T cells from melanoma patients (Figure 7B) it is intriguing to speculate that loss of function mutations in *MC-1R* resulting in a higher incidence of melanoma development or possibly a more severe disease progression might be attributed to a reduced cytolytic function of tumor-specific CD8^+^ cytotoxic T cells. Moreover, UV irradiation, which has been suggested to play a role in melanoma development, might up-regulate α-MSH/MC-1R signaling in the skin resulting in increased pigmentation as well as enhanced levels of cytotoxic CD8^+^ T cells, both involved in protection against melanoma development. In contrast, loss-of-function mutations in the *MC-1R* gene impede the UV-induced up-regulation of α-MSH/MC-1R signaling and thereby might prevent the induction of tumor protection possibly accounting for the increased melanoma susceptibility in humans with red hair and fair skin. Indeed, a larger cohort or epidemiologic studies are needed to scrutinize whether treatment of skin cancer with α-MSH or other more stable agonists of MC-1R might potentially lead to an improvement of anti-tumoral immune responses.

## Materials and Methods

### Mice

C57BL/6 mice (wt; Harlan, Borchen, Germany), C57BL/6J^e/e^ recessive yellow mice characterized by a loss-of-function mutation in the MC-1R gene due to a frameshift mutation leading to a non-functional receptor (The Jackson Laboratory, Bar Harbor, MA, USA), and OT-1 transgenic mice expressing a T cell receptor specific for ovalbumin_257–264_ (SIINFEKL) on CD8^+^ T cells (Charles River Laboratories, Sulzfeld, Germany) were used at the age of 8–12 weeks and housed under specific pathogen-free conditions. All experiments were performed according to institutional regulations (A55/2002 and G34/2005; office for the environment, nature and municipal affairs North Rhine-Westfalia, Germany).

### Alpha-MSH Stimulation

Purified murine or human CD8^+^ and CD4^+^ T cells were stimulated three times per day for 2 days with either PBS or α-MSH (10^−9^ molar). Subsequently, T cells were washed five times with PBS to remove residual α-MSH and subjected to RNA isolation, FACS-analyses, kill assays or were used for adoptive cell transfer experiments.

### Reverse Transcription (RT) and Real-Time Quantitative PCR

RNA was extracted from PBS or α-MSH treated CD4^+^ or CD8^+^ T cells using RNeasy columns (Qiagen, Hilden, Germany) and cDNA was synthesized from 1 µg of total RNA with the Reverse Transcription System (Fermentas, St. Leon-Roth, Germany). Real-time quantitative PCR was performed as described previously [69], [70] using an ABI PRISM 7300 cycler (Applied Biosystems, Darmstadt, Germany) and TaqMan gene expression assays for murine as well as human β-actin, granzyme A, granzyme B, Fas (CD95), CTLA-4 (CD152), and perforin (Applied Biosystems). All mRNA levels reported in this manuscript are normalized to β-actin.

### Histology

Haematoxylin and eosin stainings (H&E) of paraffin embedded sections (3–6 µm) from mouse skin was performed according to standard methods.

### Immunofluorescence Staining

Immunofluorescence staining was performed on cryostat sections (3–6 µm) according to standard methods [71]. Slides were incubated in the appropriate dilutions of antibodies (anti-murine CD8, clone 53–6.7 or anti-murine granzyme B, clone 16G6; both purchased from NatuTec, Frankfurt, Germany) or an isotype control (NatuTec) and subsequently incubated with an Alexa Fluor 488- or Alexa Fluor 568-labeled secondary antibody (Invitrogen, Karlsruhe, Germany). Slides were examined using an Olympus BX61 microscope and the Analysis software (Olympus, Münster, Germany). TUNEL staining was performed using the *in situ* cell death detection Kit (Roche, Grenzach-Wyhlen, Germany).

### Cell Preparation and Flow Cytometry

Single cell suspensions of mouse lymph nodes and spleens were prepared as described previously [72] and CD4^+^ as well as CD8^+^ T cells were separated by negative enrichment using magnetic beads (Miltenyi Biotech, Bergisch Gladbach, Germany). Expression of cell surface markers was analyzed by standard multi-color flow cytometry on a FACScalibur™ flow cytometer with CELLQuest™ software (BD, Heidelberg, Germany). Cells were stained with anti-granzyme B (clone 16G6), anti-CD8 (clone 53-6.7), anti-CD4 (clone RM4-5) and anti-MHC class I (clone 28-14-8). Anti-mouse MC-1R was purchased from Alpha Diagnostic, San Antonio, TX, USA. Isotype-matched control antibodies were included in each staining.

### Ethics Statement

After written informed consent was obtained PBMC were isolated from 100 ml of heparinized blood from healthy volunteers and melanoma patients by Ficoll gradient centrifugation according to standard methods (Ficoll reagent was purchased from PAA, Pasching, Austria). Subsequently, CD8^+^ T cells were sorted using the RosetteSep CD8^+^ T cell enrichment kit (Stemcell Technologies, Köln, Germany). All experiments were approved by the ethical committee of the University of Münster Medical School (2007-215-f-S).

### Sequencing and MC1R Mutation Analysis

Genomic DNA was isolated from PBMC by standard methods. The coding sequence of the *MC1R* gene was amplified using three primer pairs: a) forward 5′-GCAGCACCATGAACTAAGCA-3′ and reverse 5′-TGCAGGTGATCACGTCAATG-3′; b) forward 5′- GGAGCAACGTGCTGGAGAC-3′ and reverse 5′-CAGGATGGTGAGGGTGACA-3′; c) forward 5′-GTGCTGTACGTCCACATGCT-3′ and reverse 5′-GGTCACACAGGAACCAGACC-3′. Mutation analysis was performed by direct sequencing using the Big Dye Terminator Protocol. Sequences were directly read out in an ABI 3700 sequencer (Applied Biosystems). Data analysis was performed using Chromas Version 1.43 (Conor Mc Carthy, Griffith University, Brisbane, Queensland, Australia).

### Contact Hypersensitivity (CHS)

Mice were sensitized by painting 100 µl of 0.5% DNFB, in acetone/olive oil (4/1) on the shaved back. For elicitation of CHS responses, 12 µl of 0.3% DNFB were painted on both sides of the left ear on day five. CHS was determined by the degree of ear swelling of the hapten-exposed left ear compared to the ear thickness of the not-challenged right ear and measured with a micrometer (Mitutoyo, Tokyo, Japan). Mice that were ear challenged without prior sensitization served as negative controls. In some experiments 5×10^6^ PBS- or α-MSH treated CD4^+^ or CD8^+^ T cells were injected intravenously into DNFB-sensitized mice 24 h prior to ear challenge.

### Cytotoxicity Assay

Cytotoxicity assays were performed as described [73]. In brief, CD8^+^ T cells from wt and C57BL/6J^e/e^ mice were stimulated with PBS or α-MSH, and co-cultured for 9 d with bone marrow derived DC (bmDC) from Balb/c mice whereas bmDC were renewed every 3 d. Subsequently, L1210/3 leukemia target cells/ml were loaded with ^51^Cr and 1×10^4^ target cells were added to indicated concentrations of CD8^+^ T cells. Six hours later culture supernatants were harvested, mixed with OptiPhase Supermix (Perkin-Elmer, Waltham, MA, USA) and ^51^Cr release was measured using the Microbeta Plus liquid scintillation counter (Perkin-Elmer).

### Generation of bmDC

BmDC were generated as described [73]. Briefly, single cell suspensions were prepared from bm of wt mice and cultured in the presence of 150 U/ml GM-CSF and 75 U/ml IL-4 (R&D Systems, Wiesbaden, Germany). At day 9 of culture DC were either pulsed with SIINFEKL peptide or DNBS for 2 h at 37°C or left untreated and co-cultured with PBS- or α-MSH stimulated CD8^+^ T cells.

### Mixed Lymphocyte Reactions

CD8^+^ T cells (1×10^6^/ml) were purified from peripheral lymph nodes of OT-1 mice, stimulated with α-MSH (10^−9^ molar) for 48 h and cultured in triplicates in 96-well-round-bottom plates, in a final volume of 200 µl. DC were generated from bone marrow of wt mice, pulsed with SIINFEKL or HSV-1 gB peptides (1 µg/ml) for 2 h at 37°C and added to the OT-1 T cells in a 1/40 ratio. Mixed lymphocyte reactions were cultured for 72 h in a final volume of 200 µl. Proliferation of OT-1 T cells was assessed by ^3^H-thymidine incorporation (1 µCi/well) added for the last 12 h of the experiment.

### Inoculation of Tumor Cells and Tumor Therapy

B16-F10 mouse melanoma cells or B16-F10 melanoma cells expressing the chicken ovalbumin gene (B16-OVA) [74] were injected subcutaneously into the right flank of wt or C57BL/6J^e/e^ mice. In some experiments, at indicated time points after inoculation of tumor cells recipients were injected intravenously with 5×10^6^ PBS or α-MSH stimulated CD8^+^ cells. Tumor growth was assessed three times per week with a digital caliper (Mitutoyo) and mice with tumors exceeding 1000 mm^3^ were sacrificed according to animal care regulations.

### Alpha-MSH Radioimmunoassay

Alpha-MSH was quantified in culture supernatants as well as in the serum or tumor lysates from mice using the α-MSH radioimmunoassay (IBL, Hamburg, Germany) according to the manufacturer's instructions. Briefly, the α-MSH concentration was assessed by competitive radioimmunoassay using an antiserum to an α-MSH-albumin conjugate. Alpha-MSH in the samples competed with ^125^I-labelled α-MSH in binding to the antibodies. Antibody-bound ^125^I-α-MSH was separated from the free fraction using polyethylene glycol precipitation. The radioactivity of the precipitates was measured by scintillation counting. Importantly, the antiserum used in this kit was directed against the C-terminal part of the α-MSH molecule and showed no cross-reactivity with adrenocorticotropic hormone.
